# High-speed duetting – latency times of the female acoustic response within the bush-cricket genera *Leptophyes* and *Andreiniimon* (Orthoptera, Phaneropteridae)

**DOI:** 10.3897/zookeys.750.23874

**Published:** 2018-04-16

**Authors:** Klaus-Gerhard Heller, Olga Korsunovskaya, Bruno Massa, Ionuț Ștefan Iorgu

**Affiliations:** 1 Department of Biology, Humboldt-Universität zu Berlin, Berlin; postal address: Grillenstieg 18, 39120 Magdeburg, Germany; 2 Department of Entomology, Faculty of Biology, Moscow State University, 119234, Moscow, Leninskie Gory 1-12, Russia; 3 Department of Agricultural, Food and Forest Sciences, University of Palermo, Viale Scienze Bd 4A, 90128 Palermo, Italy; 4 “Grigore Antipa” National Museum of Natural History, Kiseleff Blvd. 1, Bucharest, Romania

**Keywords:** Phaneropterinae, katydid, female acoustic signals, duet, stridulatory movement

## Abstract

To find a mate, male and female bush-crickets of the family Phaneropteridae typically engage in duets. The male sings and the female responds. For mutual recognition, the amplitude pattern of the male song and the species-specific timing of the female response have been shown to be very important. In the seven studied species, belonging to the genera *Leptophyes* and *Andreiniimon*, these duets are extremely fast and nearly completely in the ultrasonic range. The females produce very short sounds by fast closing movements of the tegmina. They respond with species-specific delays of 20 to 150 ms after the beginning of the male song. The different latency times are probably not important for species recognition, since in sympatric species they are quite similar.

## Introduction

In bush-cricket (katydid) species of the family Phaneropteridae (or subfamily Phaneropterinae, depending on author) not only the males produce songs, but typically the females respond acoustically to these signals (for a review, see Heller et al. 2015). Exchanging sounds, males or females or both approach the other sex phonotactically. Using this bi-directional communication system, the insects may meet faster and safer than in a system where the male signals continuously without knowing if there is an interested receiver. However, here not only the female must recognise the male song; the male must also know which sounds represent answers to his song. While the male songs exhibit species-specific temporal patterns of amplitude modulation and sometimes even last for many seconds (e.g., *Ducetia japonica* group; Heller et al. 2017), the female response is typically a short click or a series of clicks. Sometimes, these sounds are difficult to discriminate from similarly structured environmental noise.

At the end of the last century, three groups of scientists studied simultaneously and independently this communication system (Heller and Helversen 1986; Robinson et al. 1986; Zhantiev and Korsunovskaya 1986) and discovered that the males use ‘auditory time windows’ for recognition. A female response must be heard within a certain time interval after the male song, otherwise it is disregarded. As discussed by Heller and Helversen (1986), in this way the male increases his signal to noise ratio for the detection of female signals and, at the same time, these windows might be used for species recognition. Different species have indeed different female latency times and different male time windows (Heller and Helversen 1986), but the tested species were not closely related. In the meantime, many different male song patterns have been detected, and even cryptic species, differing mainly in male song (e.g., Heller and Reinhold 1992: *Poecilimon paros*; Iorgu 2012b: *Isophya dochia*) have been found. The acoustic behaviour of the females, however, has received less attention. According to some studies, auditory time windows do not seem to be important for female species recognition. The females of all four *Barbitistes* species tested have quite similar latency times (Stumpner and Meyer 2001). Also in the subgenus Hamatopoecilimon, all studied species were similar in this respect (Heller et al. 2011). In both groups, the male songs differ clearly and contain marker syllables to trigger the female response. In the species-rich genus *Isophya*, however, the situation is different. Some species also possess trigger elements in the male song, and the females respond shortly after these markings (e.g., *I. bucovinensis*; Iorgu et al. 2017, see Iorgu 2012b for similar examples), but in others the female response seems to be triggered by any syllable of the male song (e.g., *I. sicula*; Orci et al. 2010), and in still others the female response occurs invariably with a fixed and relatively long delay after the end of the male song (e.g., *I. stepposa*; Zhantiev and Korsunovskaya 1986).

In the present paper we focus on the genus *Leptophyes* Fieber, 1853 and the monotypic genus *Andreiniimon* Capra, 1937. Both genera are closely related to *Isophya* (Ullrich et al. 2010). *Andreiniimon* and most of the nine European *Leptophyes* species have simple songs, which are quite similar to each other (see review in Kleukers et al. 2010; Ingrisch and Pavicevic 2010; the species *L. asamo* Pavićević & Ivković, 2014 is considered as a presumed synonym of *L. punctatissima*, following Chobanov et al. 2016). However, some species occur sympatrically over large parts of their range, so that acoustical discrimination would seem to be useful. Within the genus, large differences in mating behaviour have been observed [e.g., in spermatophore size and sperm number (Vahed and Gilbert 1996) and sexual refractory period (Vahed 2007)], which may theoretically also affect the acoustic communication between the sexes. The male calling songs are known from all species; the female response behaviour, however, is so far known from only three. We present additional data on these three species (*A. nuptialis, L. albovittata, L. punctatissima*) and describe the female acoustic behaviour of another four species (*L. discoidalis*, *L. laticauda*, *L. lisae*, *L. sicula*) for the first time.

## Material and methods

The female response behaviour was studied in the laboratory using virgin females, collected as nymphs (or from a laboratory culture, only some *L. punctatissima* females). We studied the following species (number of females studied in brackets): *Andreiniimon nuptialis* (3), *Leptophyes albovittata* (4), *L. punctatissima* (6), *L. discoidalis* (1), *L. laticauda* (5), *L. lisae* (4), *L. sicula* (1). The duets were recorded – mostly in the evening – using a Racal store 4-D tape recorder (Racal Electronics plc, Weybridge, United Kingdom) and modified tape-recorder Yupiter 202-Stereo (Komunist Works, Kiev, USSR/Ukraine), with microphones Brüel & Kjær 4133 and 4135 (B&K, Nærum, Denmark; frequency response flat up to 40 and 70/100 kHz respectively; distance to microphone 50 to 100 mm). *Leptophyes discoidalis* was recorded using a digital audio recorder EDIROL R-09HR (Roland Corporation US, Los Angeles, USA; frequency response flat 20 Hz – 40 kHz; sampling rate 96 kHz). Wing movements were registered by an opto-electronic device (Helversen and Elsner 1977; modified as in Heller 1988). Additionally, duets were recorded directly on computer using a sound card (M-Audio transit; M-Audio, Cumberland, Rhode Island, United States; sampling rate was mostly set to 64 or 96 kHz) and the microphones Uher M 645 (Munich, Germany) and Sony ECM-121 (Sony, Tokyo, Japan). A male and a female were placed separately into two plastic tubes (*Drosophila* tubes 28.5×95 mm, Biosigma, Cona (VE), Italy) standing side by side, with one microphone placed inside or on top of each vial. Both microphones typically picked up male and female sounds, but with different amplitudes. In a comparison of these signals no distortions in time or frequency domain were found. The output of each microphone was registered as one track of a stereo recording. After digitising the songs on a computer, oscillograms (after high pass filtering, typically around 1 kHz) and sound analyses were made using the programs Turbolab (TL 4.0, Stemmer, Puchheim, Germany), Amadeus (Amadeus II, Martin Hairer, http://www.hairersoft.com) and Audacity (Audacity 2.1.0; http://audacity.sourceforge.net) on Apple. Each data point is based on not less than 10 independent measurements of latency time of a female (except for one female of *L. lisae* with only seven measurements and two females of *A. nuptialis*, with five measurements), given as mean ± standard deviation (SD). For the frequency measurements given in the Results, recordings made with a digital bat-detector (Pettersson D1000X; Pettersson Elektronik AB, Uppsala, Sweden; frequency response flat 5–235 kHz: sampling rate 100 or 192 kHz) and with the R-09HR (see above) were evaluated using fast fourier transformation (FFT) analysis with hanning window, 512 points per frame, from one frame or the mean of several overlapping frames.

*Song* — Latency time: interval between beginning of male song/song model to beginning of female response. Calling song: song produced by an isolated male. Syllable: the sound produced by one complete up (opening) and down (closing) stroke of the wing. Echeme: a first-order assemblage of syllables. Impulse: a simple, undivided, transient train of sound waves (here: the highly damped sound impulse arising as the impact of one tooth of a stridulatory file).

Measurements of body and spermatophore mass follow McCartney et al. (2009).

## Results

The females of all species studied here (*Leptophyes punctatissima* (Bosc, 1792), *L. albovittata* (Kollar, 1833), *L. laticauda* (Frivaldszky, 1868), *L. discoidalis* (Frivaldszky, 1868), *L. lisae* Heller & Willemse, 1989, *L. sicula* Kleukers, Odé & Fontana, 2010, *Andreiniimon nuptialis* (Karny, 1918)) responded to male songs with very short signals consisting of one to three loud impulses and occasionally of some more soft ones. Rarely, up to five loud impulses were observed. In all species studied in this respect, the loud impulses were produced by a closing movement of the tegmina. The peak of the carrier frequency of the females’ responses was mostly similar to that of the males, with the notable exception of *A. nuptialis* (Table 1). Timing and latency of the responses are described below.

**Table 1.** Peak frequencies of male and female song and body and spermatophore mass in European *Leptophyes* and *Andreiniimon* species.

**Species**	**Male (kHz)**	**Source**	**Female (kHz)**	**Source**	**Body mass male/female (mg)**	**Spermato-phore mass (% male body mass; n)**	**Source**
*L. punctatissima*	40	F	40	F	172/302	5.8; 2	B
*L. albovittata*	50–57	C, G, H	67	D	124/255	6.3; 9	B
*L. laticauda*	20–23	C, D	22	D	423/668	24.6; 21	B
*L. discoidalis*	32–35	A, E	35	A	–	–	–
*L. lisae*	27(–30)	C, D	30	D	177/297	4.5; 6	B
*L. sicula*	29	A	30	A	203/354	–	A
*A. nuptialis*	61	C, D	44	D	354/494	9.8; 2	B

A this paper B Dagmar von Helversen, unpublished C Heller (1988) D Heller et al. (2015) E Iorgu (2012a) F Robinson et al. (1986) G Zhantiev and Korsunovskaya (1986) H Zhantiev and Korsunovskaya (2015)

### *Leptophyes punctatissima*

**Specimens studied**: 5 females, GREAT BRITAIN: laboratory culture, 1 ix–31 x 1983, leg. C. Hartley; 1 female, GERMANY: Nürnberg (49°27'N, 11°3'E), 1 x–31 xii 1987, leg. K. Reinhold.

**Figure 1.**
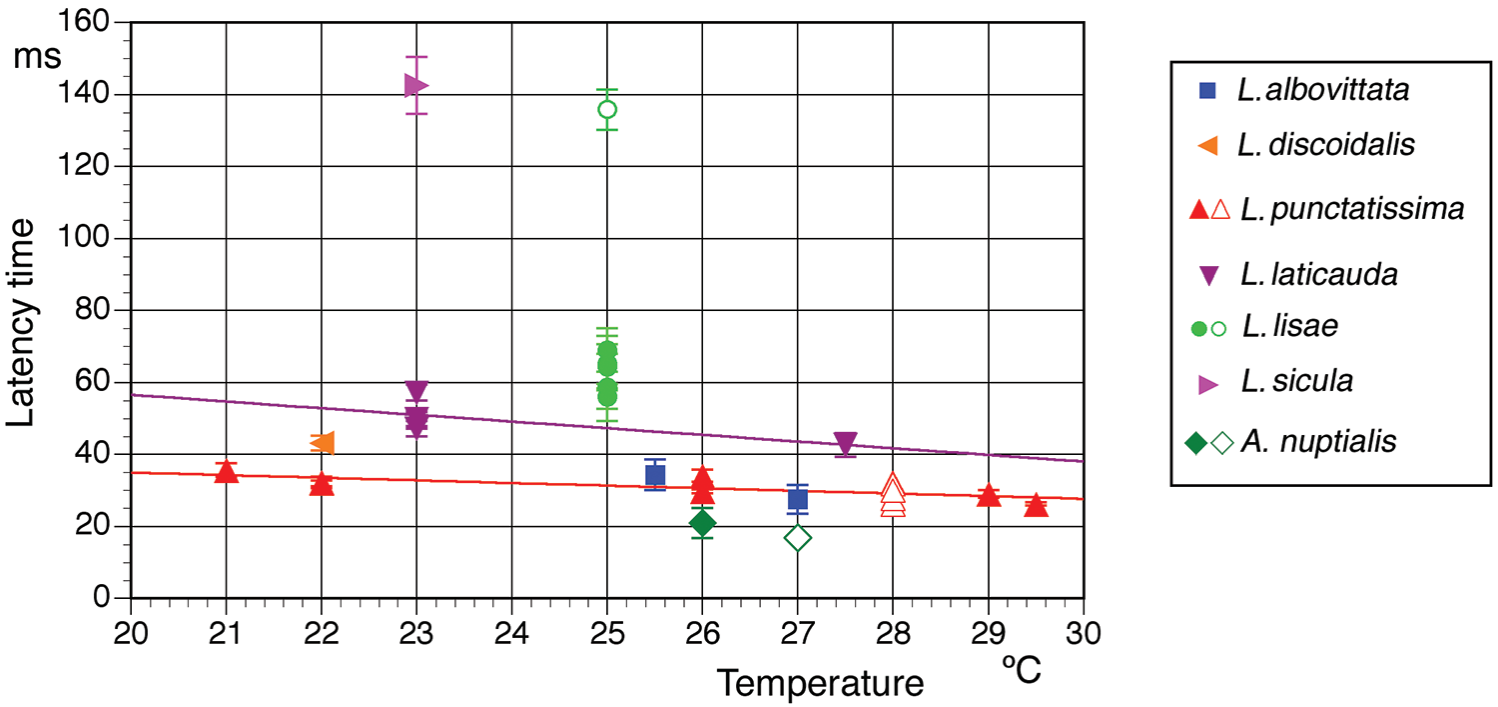
Latency times of the female response. The times refer to the beginning of the male song with temperature in European *Leptophyes* species and *Andreiniimon nuptialis.* Open symbols: *L. punctatissima* from Robinson et al. 1986; *L. lisae*, see text; *A. nuptialis* from Heller and Helversen 1986. Error bars indicate SD, regression lines based on own data (for details, see text).

**Figure 2.**
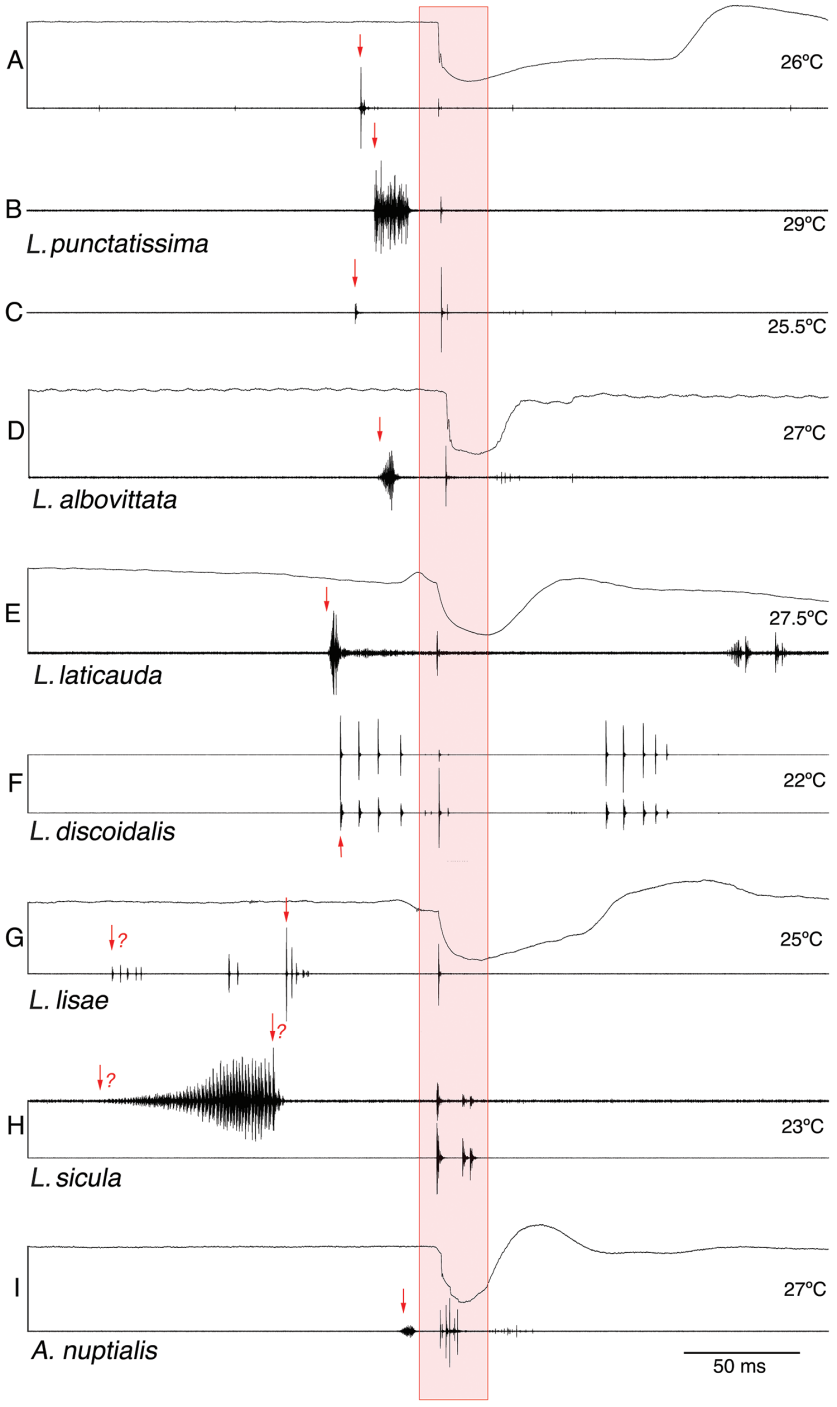
Oscillograms of male-female-duets. Stridulatory movement of female *Leptophyes* and *Andreiniimon* together with male-female duet (sound **A, D, E, G, I**) or male-female duet, sound only (**B, C, F, H**). Oscillograms of stridulatory movement and song [synchronous registration of left tegmen movement and sound (upper line: upward deflection represents opening, downward closing; lower line: sound)]. **A–C** female reaction to model of male song **D** female reaction to heterospecific male song (see text) **E–I** female reaction to conspecific male song. Female responses in box highlighted in red, proposed trigger point marked with red arrow.

The male calling song consists of single, short syllables presented at intervals of several seconds. The acoustical response behaviour of the female was studied intensively by Hartley and Robinson (1976), Robinson (1980), Robinson et al. (1986) and Zimmermann et al. (1989). Robinson et al. (1986) gave detailed information about the latency times. Here we add data on the temperature dependency of this behaviour (Fig. 1; f(x)=-0.7299x+49.53; r^2^=0.6336) and demonstrate that the female reaction can be elicited by crude, click-like models of the male song. Females responded very reliably to fingernail snips (Fig. 2A) – acoustically an impulse of about 1 ms or less in duration with most energy in the ultrasonic range. This behaviour is well known in the laboratories working with this species. Rectangularly-modulated pulses of white noise with a duration of 15 ms were responded to with about the same latency, measured from the beginning of the pulse (Fig. 2B).

### *Leptophyes albovittata*

**Specimen studied**: 1 female, GREECE: N. Drama, valley of river Nestos, above Paranestion, (41°17'N, 24°29'E), 5 m, 17 vi 1984, leg. v. Helversen; 3 males, 3 females, RUSSIA, Kursk distr.: Centralno-Chernozemny reserve (51°09'N, 36°26'E), 10–13 viii 1985, leg. O. Korsunovskaya.

The male calling song consists of single, short syllables presented at intervals of several seconds. The acoustic response behaviour of the female was first studied by Zhantiev and Korsunovskaya (1986, 1990, 2015). They showed that the female latency time is constant with reference to the beginning of the song. The females responded to song models independently of the duration of the model (tested from 10 ms up to 100 ms). Here we show that even very short signals (see *L. punctatissima*) are answered (Fig. 2C). It was therefore not surprising to see that calling songs of other species with the appropriate spectral properties were also answered (here *Andreiniimon nuptialis*; Fig. 2D). The female responses are produced during closing movements of the tegmina. However, during the re-opening of the tegmina soft impulses were sometimes observed (Fig. 2D).

### *Leptophyes laticauda*

**Specimens studied**: 1 male, 1 female, ITALY: Medeazza near Trieste (45°55'N, 13°25'E), 1–10 vii 1995, leg. v. Helversen; 1 male, 1 female, MONTENEGRO: Petrovac (42°12'N, 18°56'E), 9 vi 2017, leg. M. Heller; 3 females, MONTENEGRO: Lovcen pass above Kotor (42°24'N, 18°45'E), 4 vi 2017, leg. M. and K.-G. Heller; 1 male, MONTENEGRO: Zabljak (42°19'N, 16°9'30"E), 8 vi 2017, leg. M. Heller.

The male calling song of *L. laticauda* is more variable and the song units (see below) are produced in a much faster rhythm (Heller 1988; Ragge and Reynolds 1998) than in the two previously described species. Each song unit consisted sometimes of one short, compact syllable only. Sometimes, this part was followed by an isolated impulse (‘after-click’; see e.g., fig. 23G in Heller 1988). In the most often observed combination (echeme), one loud syllable (with or without after-click) was combined with a soft second syllable of variable internal structure (see male sound in Fig. 2E; see also Heller 1988; Fontana et al. 2002). Occasionally, several such soft syllables followed each other in short intervals (see description and figures in Roesti and Keist 2009). The females responded nearly exclusively in a fixed interval after the first and loudest syllable (Fig. 1). The impulses were produced during a closing movement, but in the movement track it can be observed that the female reacted a few ms earlier with a small tegmen or body movement (Fig. 2E). The latency time is slightly longer than in *L. punctatissima* and similarly, it depended on temperature (f(x)=-1.8556x+93.63; r^2^=0.502).

### *Leptophyes discoidalis*

**Specimens studied**: 1 male, 1 female, ROMANIA: Telciu (47°24'N, 24°23'E), 7 vii 2017, leg. I. Ș. Iorgu.

In contrast to all other European *Leptophyes* species, the male calling song consists not of single syllables (or very short echemes), but of a series of six up to 30 syllables (Ingrisch and Pavicevic 2012; Iorgu 2012a). The amplitude modulation of the echeme is decreasing with the loudest syllables at the beginning. The female answered after a fixed time interval after the beginning of the first syllable (=after beginning of an echeme). The female never answered to another syllable of an echeme, but only to the first syllable after a larger gap (interval between last syllable of previous echeme and beginning of the next, 817 ± 151 ms; n=10).

### *Leptophyes lisae*

**Specimens studied**: 1 male, 1 female, GREECE: N. Korinthia, southeast of Korinth (37°50'N, 23°2'E), 1–30 iv 1984, leg. E. Blümm; 1 male, 3 females, GREECE: Chios, 2 km west of Mesta (38°16'N, 25°54'E), 24 v 1995, leg. K.-G. Heller.

The male calling song consists of single syllables, but these syllables are much longer (ca. 150 ms at 20 °C; Heller and Willemse 1989) than in the species mentioned above and contain three groups of impulses. The last group is the loudest and decreasing in amplitude. The females answered with a fixed delay of about 60 ms after the last syllable group. Judging from the female reaction to the male song it would have been difficult to determine the trigger point for the response, but all three females tested answered to click-like song models with the same latency as to the beginning of the last group of impulses in the male song. Interestingly, one of these females had a bi-modal distribution of latency times. It switched irregularly between ‘normal’ response times (69±6 ms, range 58–78 ms, n=10) and much longer ones (136±6 ms, range 128–143 ms, n=10; see Fig. 1, open symbol).

### *Leptophyes sicula*

**Specimens studied**: 1 male, 1 female, ITALY: Sicily, 1–31 viii 2015, leg. Bruno Massa.

The male calling song consists of relatively long (Kleukers et al. 2010: 40–50 ms), crescending single syllables. The female answered 142.5±8 ms (range 127–160 ms; n=30) after the beginning of this syllable, or 69±7 ms (range 53–81 ms; n=30) if measured from its end.

### *Andreiniimon nuptialis*

**Specimens studied**: 1 male, 1 female, FYR MACEDONIA: 10 km w Miravci, Vardar near Demir Kabija (41°24 N, 22°9'E), 21 vi 1984, leg. D. and O. v. Helversen; 2 males, 2 females, GREECE: N. Ilia, Peloponnesos, valley of river Erimanthos, 6 km east of Koumanis (37°48'N, 21°47'E), 1–30 vi 1997, leg. K.-G. Heller.

The male calling song consists of single, very short syllables presented at intervals of several seconds. The female response occurred only about 20 ms later (see also Heller and Helversen 1986), typically consisting of several impulses. Its peak frequency was clearly lower than that of the male song (Table 1).

## Discussion

The females of *Andreiniimon* and all six *Leptophyes* species studied here responded acoustically to the male calling song with very short signals, consisting of a few sound impulses only, produced by a closing movement of the tegmina (Fig. 2). These sounds were produced after quite short latency times, less than 200 ms after the beginning of the male signal (Fig. 1).

### Latency times

Five species are grouped together with reaction times shorter than 60 ms. The fastest species, with a latency of about 20 ms or lower, was *Andreiniimon nuptialis.* It is thus the species with the fastest known duet in insects world-wide (see Bailey and Hammond 2003; their latency times refer to the end of the song). Only slightly slower are *L. punctatissima* and *L. albovittata* with latencies between 20 and 40 ms. In these two species the male calling songs and the peak frequency of the male and female are similar making acoustic discrimination difficult. Western *L. punctatissima* and eastern *L. albovittata* populations overlap in large parts of Central and Eastern Europe – both species are typically not found syntopically – but their ecological separation is strong enough to make acoustic differentiation unnecessary. The range of a fourth species, *L. laticauda*, lies nearly completely inside the area of one of the two others or even in that part where these overlap. Concerning the latency of the female response, it is slower than the others, but probably not enough to prevent misidentifications completely, at least by males of the faster species. The phonotactic reaction of males of *L. punctatissima* stops at about 45 ms (Robinson et al. 1986), so they might be attracted by fast *L. laticauda* females. However, the different spectra (Table 1) and the much faster rhythm in which the syllables/echemes of *L. laticauda* are produced will make acoustical problems unlikely. The long intervals between the syllables in *L. punctatissima* are probably not attractive for *L. laticauda* females. The fifth species, *L. discoidalis*, seems also to occur sympatrically with three others (*L. albovittata, L. boscii, L. punctatissima* and perhaps even *L. laticauda*; see Kleukers et al. 2010). Its females reacted even faster than *L. laticauda*, but the structure of the male calling song is very different (see below).

The last two species, *L. lisae* and *L. sicula*, have longer latency times than the five discussed above. Both are morphologically most similar to each other and then to *L. punctatissima*, but none occurs sympatrically with any other according to present knowledge. Otherwise, the three species would present a nice example of the importance of latency times. Males of *L. punctatissima* will certainly not accept the slow *L. lisae* females (see Zimmermann et al. 1989), and for *L. lisae* males the same might be assumed concerning *L. sicula* females. However, especially for *L. sicula* the possible mechanism of triggering the female response has to be discussed.

Why do the females use such short latency times? If the male phonotactically approaches a responding female, he may prefer the nearest one. The sound needs three milliseconds to travel one metre, so by answering rapidly a female can get an advantage. In *L. punctatissima* the males even did not walk towards a female whose response they received later than 55 ms and with an intensity lower than 50 dB SPL. Successful duetting started only at distances lower than four metres (Zimmermann et al. 1989). Such narrow male time windows, however, exist probably only in species with extremely short female latency times (see fig. 8, Heller and Helversen 1986). If latencies become larger, variability will increase, assuming a similar coefficient of variation.

### Song recognition

In species in which the females respond to song models which differ from the species-specific song pattern, artificial signals nevertheless contain the information necessary for species recognition and for triggering the female acoustic response. The females of some *Leptophyes* species do not seem to be very selective concerning species recognition. They responded to signals much shorter and much longer than the song (see above, Zhantiev and Korsunovskaya 1986). The same non-selective behaviour was also found in *Poecilimon ornatus*, a species from a related phaneropterid genus (Heller et al. 1997). However, this does not necessarily mean that all species with females responding to the beginning of the male song are non-selective. They may evaluate previous signals and then decide to answer to the next signal. In a stereotyped form this situation is found in species where the male song contains trigger syllables (e.g., in *Ancistrura nigrovittata*, see Dobler et al. 1994). Also in non-European *Leptophyes* species male calling songs with distinct trigger syllables have been described (e.g., *Leptophyes helleri*; Sevgili 2004). In a less stereotyped form, the female may use the intervals between male songs. The longer and the more variable the intervals between the male songs are, the less likely is this effect. It is certainly weak in *L. punctatissima* and *L. albovittata*, but may be more important in *L. laticauda* and, of course, in *L. discoidalis*. In some recordings of *L. discoidalis* made by Ingrisch (recording 0373 in Cigliano et al. 2018) and in some echemes studied here, the first syllable of an echeme is separated from the rest by an unusually long interval, perhaps the first step towards a real trigger syllable. In principle, *L. lisae* could also belong in this category with the first parts of the male syllable used for evaluating. However, the females unexpectedly answered to click-like song models. Possibly they switched to a general ‘answering mode‘ after having heard some male songs. The long latency times observed irregularly in one female could indicate that she sometimes ‘assumed’ to have heard the first impulses of a syllable and is able to adapt to this situation. Although this unusual timing of *L. lisae* looks similar to the reaction of *L. sicula*, this species may use a completely different triggering process. In contrast to most other *Leptophyes* species with known male signals, it has a song with crescending syllable beginning, making exact timing of the female difficult. Depending on distance and/or noise, a female cannot easily recognise the start of the male syllable. On the other side, the end of the relatively long male syllable is clearly marked, serving as a better trigger point. Responding with a constant interval after the end of the male song was proven in *Isophya stepposa* by using song models (Zhantiev and Korsunovskaya 1986).

Of course, more studies are necessary to understand the differences in the female response behaviours between the species. There is, however, no evidence that the mating behaviour affects the acoustical response (see Table 1). For example, *L. laticauda* with spermatophores of ca. 25–30% male body weight and *L. punctatissima*, *L. albovittata* and *L. lisae* with spermatophores of 4–8% male body weight (Table 1; similar data also in Vahed 1994) do not group into acoustical response behaviours according to spermatophore nor body mass (Table 1). The same is true for carrier frequency. The large *L. laticauda* had clearly the lowest peak frequency, but *A. nuptialis*, next in body mass (Table 1), had the highest peak frequency.

So neither sexual selection (as far as the known differences in mating behaviour are concerned) nor species recognition play an easily understandable role for the evolution of female latency times. In both contexts male calling songs are probably more important. However, for the coexistence of different species ecological adaptations should not be underestimated. *Leptophyes punctatissima* and *L. albopunctata*, for example, occur sympatrically, but not syntopically over large parts of Europe (Kleukers et al. 2010) with nearly identical acoustical communication systems.
